# Commentary: Exploring the gut microbiome and immunological landscape in kidney cancer: a Mendelian randomization analysis

**DOI:** 10.3389/fimmu.2025.1506205

**Published:** 2025-03-05

**Authors:** Peiqin Zhan, Wujie Chen, Junhao Chen, Junxian Zhao, Mingxia Ding, Shi Fu, Jiansong Wang

**Affiliations:** ^1^ Department of Urology, The Second Affiliated Hospital of Kunming Medical University, Kunming, Yunnan, China; ^2^ Department of Urology, 920th Hospital of Joint Logistic Support Force, Kunming, Yunnan, China

**Keywords:** gut microbiome, immunological landscape, kidney cancer, mendelian randomization analysis, commentary

## Introduction

We read with great interest the article by Lv et al., titled “Exploring the gut microbiome and immunological landscape in kidney cancer: a Mendelian randomization analysis” (1). In this study, the authors applied Mendelian Randomization (MR) to explore the causal relationships between immune cells, gut microbiota, inflammatory factors, and kidney cancer (KC), uncovering several immune phenotypes, inflammatory proteins, and gut microorganisms significantly associated with kidney cancer. Their work offers valuable insights into potential biomarkers and therapeutic targets for KC. We greatly admire the accomplishments of the research team, especially in using MR analysis to establish associations between these biomarkers and kidney cancer. However, We believe the study could be further improved in several aspects.

## Discussion

First, regarding the outcome data, the authors used an outdated kidney cancer dataset. Specifically, they employed the FinnGen R10 version for KC data, but as of this year, the FinnGen database has been updated to version R11. The R11 version includes more patient and non-patient data and provides additional single nucleotide polymorphisms (SNPs), thereby enhancing the statistical power and reliability of the results. We recommend that the authors reanalyze the KC data using the latest FinnGen R11 version to ensure the conclusions’ accuracy and relevance. Furthermore, meta-analyses should incorporate KC datasets from different sources. The authors identified several immune cells, gut microbiota, and inflammatory proteins associated with KC using a conventional threshold of P < 0.05. However, cancer patients often exhibit significant outcome heterogeneity, potentially leading to inconsistent results. To enhance the reliability of the findings, we suggest conducting a meta-analysis using independent datasets from different sources as supplementary data. For example, the UK Biobank dataset (ukb-b-1316, https://gwas.mrcieu.ac.uk/datasets/ukb-b-1316/) would serve as an excellent complementary resource. Combining this independent outcome dataset with the meta-analysis can provide stronger evidence and mitigate bias from population heterogeneity.

Second, the absence of reverse Mendelian Randomization (MR) is a notable limitation of this study. The primary advantage of reverse MR is its ability to rule out the possibility of bidirectional causality. The authors primarily used forward MR to identify associations between immune cells, gut microbiota, and inflammatory factors with KC. However, some associations may be bidirectional, where these factors are not only exposures for KC but also consequences. Failure to consider bidirectional causality could result in findings that contradict the fundamental assumptions of MR. Additionally, reverse MR can be used to explore the impact of KC as an exposure on the immune system, gut microbiota, and inflammatory factors. For instance, reverse MR could reveal how the development of KC affects specific immune cell counts, alters gut microbiota composition, and leads to changes in inflammatory factors. This would provide a more comprehensive understanding of the pathophysiological mechanisms of KC and may uncover novel therapeutic targets.

Third, several immune cells, gut microbiota, and inflammatory factors were positively associated with KC outcomes in the study. Multivariable Mendelian Randomization (MVMR) analysis would be a valuable supplement. The authors identified significant associations using univariable MR, but these factors may have shared effects, making it difficult to determine which factors independently influence the outcome (2, 3). By applying MVMR, the authors could incorporate these positively associated factors into the model simultaneously and investigate which of them can independently affect KC incidence. This would help to identify more critical biomarkers or potential therapeutic targets.

Fourth, to ensure the accuracy of the results, we recommend that the authors incorporate false discovery rate (FDR), colocalization analysis, and Bayesian-weighted MR to further validate their findings. The authors conducted large-scale MR analyses, revealing several associations between immune cells, gut microbiota, and inflammatory factors with KC. Although these associations were based on traditional P-value thresholds (P < 0.05), large-scale MR studies inevitably increase the risk of false positives. Therefore, it is advisable to report results using FDR correction to minimize the risk of false positives and ensure the reliability of the findings. In addition to multiple testing corrections, the authors should consider incorporating Coloc analysis and Bayesian-weighted MR to address heterogeneity in the data, verify the robustness of the results, and enhance the study’s explanatory power. Coloc analysis evaluates whether the exposure factors (e.g., immune cells, gut microbiota, inflammatory factors) and the outcome (KC) share the same genetic variants. Given that cancer and its biomarkers may be influenced by the same genetic factors, Coloc analysis helps to determine whether the relationships between these biomarkers and outcomes are based on shared genetic backgrounds rather than causality. Using Coloc, the authors can verify which immune cells or inflammatory factors are truly causally related to KC and which may simply result from shared genetic variants, thereby reducing false positives. Furthermore, Bayesian-weighted MR assigns weights to each SNP based on its effect size, addressing inherent complexity and heterogeneity in the data. Compared to traditional MR methods, Bayesian-weighted MR offers greater flexibility and adaptability when analyzing multi-dimensional exposure factors and outcomes (4). In cancer research, the genetic diversity and complex genetic backgrounds among individuals often lead to uncertainty and bias in results. By assigning weights based on Bayesian statistical methods, Bayesian-weighted MR can more accurately assess causal relationships between SNPs and outcomes, reducing potential confounding due to effect heterogeneity.

Currently, direct research on how changes in the gut microbiota contribute to the progression of renal cancer is still in the exploratory stage, and relevant literature remains limited. However, existing studies suggest that the gut microbiota may influence tumorigenesis and progression through various mechanisms, which may also be applicable to renal cancer.For example, in a Min mouse model, the pathogenic pks-positive Escherichia coli strain (11G5) was found to significantly induce tumorigenesis while increasing both the size and number of adenocarcinomas (5). Additionally, metabolites, toxins, or molecular components produced by the gut microbiota can alter intestinal epithelial permeability or modulate the immune system. Cuiru Li et al. found that short-chain fatty acids (SCFAs) produced by gut microbiota metabolism can regulate host immune responses and inflammatory states, thereby affecting tumorigenesis and progression (6). Moreover, studies have indicated that the specific composition of the gut microbiota may lead to primary resistance to immune checkpoint inhibitor (ICI) therapy (7). In our study, we also observed a positive correlation between the gut bacterium Odoribacter splanchnicus and the incidence of renal cancer, suggesting its potential role in promoting inflammatory responses and influencing the tumor microenvironment, thereby contributing to renal cancer progression. However, some studies have reported anti-inflammatory properties of this bacterium, which contradicts our findings. This discrepancy suggests that Odoribacter splanchnicus may exert different effects depending on the type of cancer or individual variations. Therefore, further validation is required to determine the precise role of specific bacterial species, such as Odoribacter splanchnicus (8). Additionally, previous studies have demonstrated that interleukin-2 (IL-2) exerts antitumor effects by enhancing T-cell activity, but it may also promote regulatory T (Treg) cells, thereby suppressing immune responses. However, our study primarily focused on its antitumor properties while overlooking its potential immunosuppressive effects (9). Future research should further investigate the dynamic changes in specific bacterial species and inflammatory factors through animal models and clinical data to elucidate their precise impact on renal cancer.

## Conclusion

Overall, Lv et al.’s research lays an important foundation for studying KC biomarkers and exploring therapeutic targets. We hope these suggestions provide valuable guidance for the authors’ future research and promote further advancements in this field.
